# *Helicobacter pylori* strains harboring *babA2* from Indian sub population are associated with increased virulence in ex vivo study

**DOI:** 10.1186/s13099-015-0083-z

**Published:** 2016-01-12

**Authors:** Prachetash Ghosh, Avijit Sarkar, Mou Ganguly, Raghwan, Jawed Alam, Ronita De, Asish K. Mukhopadhyay

**Affiliations:** Division of Bacteriology, National Institute of Cholera & Enteric Diseases, P 33, CIT Road, Scheme XM, Beliaghata, Kolkata, 700010 India

**Keywords:** *Helicobacter pylori*, Duodenal ulcer, *BabA2*, IL-8, Adhesion molecule, Apoptosis

## Abstract

**Background:**

The *babA2* gene along with the *cagA* and *vacA* of *Helicobacter pylori* has been considered as a risk factor for the disease outcome in certain populations. This study was aimed to understand the role of *babA2* of *H. pylori* with the background of *cagA* and *vacA* in disease manifestations in Indian sub population.

**Methods:**

A total of 114 *H. pylori* strains isolated from duodenal ulcer (DU) (n = 53) and non-ulcer dyspepsia (NUD) patients (n = 61) were screened for the prevalence of these virulence markers by PCR. The comparative study of IL-8 production and apoptosis were done by co-culturing the AGS cell line with *H. pylori* strains with different genotypes. Adherence assay was performed with *babA2* positive and negative strains. Two isogenic mutants of *babA2* were constructed and the aforesaid comparative studies were carried out.

**Results:**

PCR results indicated that 90.6 % (48/53), 82 % (50/61) and 73.6 % (39/53) strains from DU patients were positive for *cagA*, *vacA*, and *babA2,* respectively. Whereas the prevalence of these genes in NUD subjects were 70.5 % (43/61); 69.8 % (37/53), and 65.6 % (39/61), respectively. Although adherence to AGS cells was comparable among strains with *babA2* positive and negative genotypes, but the triple positive strains could induce highest degree of IL-8 production and apoptosis, followed by the *cagA*^−^/*vacA*^−^/*babA2*^+^ strains and triple negative strains, respectively. The wild type strains showed significantly higher IL-8 induction as well as apoptosis in ex vivo than its isogenic mutant of *babA2*.

**Conclusion:**

PCR study demonstrated that there was no significant association between the distribution of *babA2* genotype or of triple positive strains and disease outcome in this sub population. The adherence assay showed that there was no significant difference in the extent of adherence to AGS cells among *babA2* positive and negative strains. But the ex vivo study indicated that the triple positive or even the *babA2* only positive strains are involved in increased virulence. The wild type strains also exhibited increased virulence compared to the *babA2* mutant strains. This inconsistency demonstrated that bacterial genotype along with host genetic polymorphisms or other factors play important role in determining the clinical manifestation of *H. pylori* infections.

## Background

*Helicobacter pylori* is a Gram-negative, genetically diverse spiral bacteria that infects more than half of the population worldwide [1]. Infection with *H. pylori* is associated with duodenal ulcer (DU) or gastric ulcer, gastritis, and gastric adenocarcinoma [2]. About 65–70 % of the Indian population is infected with *H. pylori* [3, 4]. 15–20 % of overall infected population develop gastric or duodenal ulcer and less than 1 % develop gastric adenocarcinoma [5].

The genome of various *H. pylori* strains demonstrates significant genetic diversity. Genetic variation in specific virulence genes of *H. pylori* may participate in the pathogenic process of *H. pylori* infection in the stomach, thereby contributing to the variable risk of diverse clinical outcomes. So, in addition to the host immunological factors and environmental factors, another important reason for the diverse clinical outcomes is the differences in virulence factors among *H. pylori* strains [6].

Cytotoxin-associated gene (*cagA*) was the first reported gene that varies in *H. pylori* strains and is considered as a marker for the presence of the *cag* pathogenicity island (*cag* PAI), which includes a number of other genes associated with increased virulence [7–9]. About one-half to two-thirds of US and European strains carry the *cag* PAI and is associated with overt disease. In contrast, *cag* PAI is distributed among most of the Asian strains, irrespective of disease status. In India the asymptomatic individuals also carry the *cag* PAI [10]. It was found that most of the *H. pylori* strains in Indian subcontinent have *cagA* typeA and any particular type of *cagA* is not associated with disease outcome [11].

Some *H. pylori* strains can produce vacuoles in epithelial cells by the action of VacA protein which consists of a signal region (*s1* or *s2*) and a middle region (*m1* or *m2*). Strains harboring the *s1m1* mosaic combination are said to be more cytotoxically potent than the strains with *s1m2* genotype, while *s2m2* strains do not secrete vacuolating cytotoxin [12]. In Western countries, *H. pylori* strains with *s1m1* genotype usually also carry the *cag* pathogenicity island (*cag* PAI) and are more significantly associated with the disease than those strains which don’t have this *cag* PAI [7]. In Indian population, the strains with *s1m1* genotype are predominant [13]. It concludes that the diseases are multi-factorial and thereby we need to identify other bacterial virulence factors which play important role in diseases manifestation.

It is generally known that bacterial adherence to the gastric epithelium is the first critical stage of colonization by *H. pylori* in the human stomach [14]. The blood group antigen binding adhesin (BabA) is one of the major outer membrane proteins of *H. pylori* that binds to the fucosylated Lewis^b^ blood group antigens on the gastric epithelium and plays a key role in facilitating bacterial colonization to the stomach [15, 16]. It has been hypothesized that the adherent bacteria may be more successfully able to transfer their products to the host cells. Thus, a higher bacterial density coupled with more capable delivery of bacterial products to the host cells may provoke a stronger inflammatory response. It has been already shown that *H. pylori* is able to commence nonspecific immune responses [17]. *H. pylori* has various adhesins, including blood group antigen binding adhesin (BabA), which binds to the Lewis B (Le^b^) antigen [15, 18]. *H. pylori* adhesin-mediated colonization is multi-factorial and no single adhesin may be essential, although adherence of this bacteria to the gastric epithelium is a compulsory early step in colonization and renders *H. pylori* 100–1000 times more resistant to antibiotics than the non-adherent ones [19]. Blood group antigen binding adhesin is encoded by a polymorphic gene named *babA2*, while the *babA1* allele is non-functional. Gerhard et al. [20] in 1999 first demonstrated a positive association between a *babA2* carrying strain and duodenal ulcer (DU) and gastric cancer (GC). Subsequently, a series of studies of the association between *babA2* gene and peptic ulcer diseases and GC have been performed, but with inconsistent or conflicting conclusions [21–24]. However, in Asian countries, most of the strains are *babA2* positive irrespective of disease status [25, 26]. These data clearly indicates that the frequency of *H. pyloribabA2* gene may vary geographically and their associations with disease outcome also vary accordingly.

Although Indian subcontinent constitutes about the 1/5th of the world population, but there was not much studies about the role of *babA2* gene in disease outcome from this subcontinent. The contradictory conclusions from previous studies and the inadequate information from India provided the impetus to study (1) the clinical relevance of the *babA2* gene by examining its association with *H. pylori* virulence-associated genes and with clinical outcome as well as (2) the comparative analysis of IL-8 production and apoptosis by co-culturing the AGS cell line with Indian *H. pylori* strains with variant genetic makeup.

## Results

A total of 185 subjects underwent for endoscopy from which 114 *H. pylori* strains were isolated. The biopsies were divided into two groups according to their clinical symptoms: duodenal ulcer (DU) and non ulcer dyspepsia (NUD). Subjects with abdominal discomfort, acidity, loss of appetite but no frank ulceration were considered as non-ulcer dyspepsia (NUD) but those have endoscopically visible duodenal ulceration were considered as duodenal ulcer (DU) patients. The strains were isolated from 53 DU patients (male = 54 %, median age = 62 years, range 28–85 years) and 61 NUD patients (male = 52 %, median age = 67 years, range 27–87 years). The genomic DNA was isolated from these strains and was used for further downstream PCR-based analysis.

### Prevalence of *babA2, cagA* and *vacA*

**Fig. 1:**
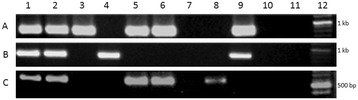
Genotyping of *babA2* gene in Indian *H. pylori* isolates. The images shown are from a representative gel electrophoresis of a PCR amplification product of *babA2* gene from Indian isolates and J99 control strain with* A* nthu_babA2F and babA2R primers [26],* B* babA2F and babA2R primers [20], and* C* babA2F and babA2R607 primers [27]. Where *lane 1* positive control, *lanes 2–10* clinical isolates, *lane 11* negative control, and *lane 12* DNA ladder

The integrity and specificity of these isolated *H. pylori* DNAs were confirmed by PCR amplification of the *urease* gene, which yielded an expected 480 bp amplicon from all the DNAs (data not shown). The distribution of *babA*2 gene in the 114 Indian *H. pylori* strains were studied first using the PCR-based genotyping. The fragment of *babA2* was amplified primarily by using forward primer nthu_babA2F [26] and reverse primer babA2R [20]. PCR assay with these primers yielded an amplicon of 832 bp (Fig. 1A) and 60.5 % (69/114) strains produced positive amplicon for *babA2* gene. Another two sets of primers (namely babA2F and babA2R; babA2F and babA2R607) were used to amplify the *babA2* gene from the strains that did not give positive amplicon with the primary set of primers [20, 27]. PCR with these two new combination of primers yielded amplicon of *babA2* fragment (832 and 607 bp respectively) from 3 (72/114, Fig. 1B) and 5 (77/114, Fig. 1C) more clinical isolates, respectively. Finally, on the basis of the combined results with these three sets of primers, our analysis depicted that 67.5 % (77/114) Indian *H. pylori* strains were positive for *babA2* gene (Table 1).

**Table 1:** Prevalence of the *babA2*, *cagA* and *vacA* genes in different disease groups

Gene	Total (n = 114)	DU (n = 53)	NUD (n = 61)
*babA2*	77 (67.5 %)	37 (69.8 %)	40 (65.6 %)
*cagA*	98 (86 %)	48 (90.6 %)	50 (82 %)
*vacA s1m1*	82 (71.9 %)	39 (73.6 %)	43 (70.5 %)

The *cagA* and *vacA* status of these 114 strains were determined using the previously described primers and protocols [5]. The *cagA* positive strains produced an amplicon of 350 bp (data not shown) and 86 % (98/114) strains were positive for *cagA* (Table 1). The strains that were negative for *cagA*, yielded a 550 bp amplicon for *cag*-empty site using the primers located in the flanking region of the *cag* PAI. *vacA s* and *m* alleles were also studied and it was shown that the most potentially toxic *vacA s1m1* allele was predominantly present in the tested strains and it showed a frequency of 71.9 % (82/114).

The *babA2* gene was determined among the two groups of patients [DU (53) and NUD (61)]. PCR results showed that 69.8 % (37/53) strains were *babA2* positive in DU and 65.6 % (40/61) *babA2* positive strains were present in NUD subjects (Table 1). The *babA2* gene was almost equally distributed among strains from DU patients and NUDs in this region. So, the prevalence of *babA2* in this region showed no significant correlation with disease outcome. Our study demonstrated that the *babA2* genotype was predominant among *H. pylori* strains irrespective of disease status.

Among the 98 *cagA* positive strains used in this study, 41 of 48 DU patients and 40 of 50 NUD individuals produced a typical ~642-bp amplicons, designated as type A by Yamaoka et al. [28] (Table 2). Six strains from DU and nine strains from NUD yielded ~756 bp amplicon in PCR using the same set of primers and considered as having *cagA* type B/D (Table 2). Two strains (one each from DU and NUD) produced a ~810 bp amplicon corresponding to type C (Table 2). No correlation with disease outcome was found with any of the *cagA* types. Sequencing analysis of the representative strains showed that all of them carried Western-CagA-specific sequences (WSS) FPLKRHDKVDDLSKV. These strains contained 1–5 EPIYA motifs in their *cagA* gene sequence. Sequence analysis also revealed that more than 80 % of the *cagA* positive strains carried 3 EPIYA motifs (A-B-C type) at the 3′ end of the gene and they were almost equally distributed between DU and NUD. Similarly, *vacAs1m1* allele was present in almost equal frequencies in DU patients and NUDs (Table 1).

**Table 2:** Prevalence of *cagA* subtypes in different disease groups

*CagA* types	DU (n = 48)	NUD (n = 50)
Type A	41 (85.4 %)	40 (80 %)
Type B/D	6 (12.5 %)	9 (18 %)
Type C	1 (2 %)	1 (2 %)

The association study of these virulence genes in each strain reveals no statistically significant **(***P* < 0.05**)** correlation between the distribution of the triple positive strains (combined presence of *cagA*, *vacA s1m1* allele and *babA2*) and disease status. Additionally, 84 isolates were classified as type 1 strains (positive for both *cagA* and *vacAs1m1*) and 61 were found as triple positive strains among them. These type 1 strains also exhibited an almost uniform distribution among DUs (40/53, 75.47 %, Table 3) and NUDs (44/61, 72.13 %, Table 3). It was found that these 61 triple positive strains distributed almost equally in DU (29/53, 54.7 %, Table 3) patients and NUDs (32/61, 52.4 %, Table 3). Among the 16 *cagA* negative strains tested, 6 (37.5 %) were found as *babA2* positive strains.

**Table 3:** Prevalence of the type 1 and triple positive strains in different disease groups

Strains	Total (n = 114)	DU (n = 53)	NUD (n = 61)
Type 1 strains	84 (73.7 %)	40 (75.5 %)	44 (72.1 %)
Triple positive strains	61 (53.5 %)	29 (54.7 %)	32 (52.4 %)

### Adherence of *H. pylori* to the gastric epithelial (AGS) cells is independent of *babA2*

Adherence of *H. pylori* to the AGS cells was performed as described in methods section and was found comparable among the strains with *babA2* positive and *babA2* negative genotypes (data not shown). It was also observed that there was significantly no difference in the ability of adherence between the wild type and isogenic *babA2* mutants (data not shown).

### Triple positive strains show greater IL-8 induction in AGS cells

**Fig. 2:**
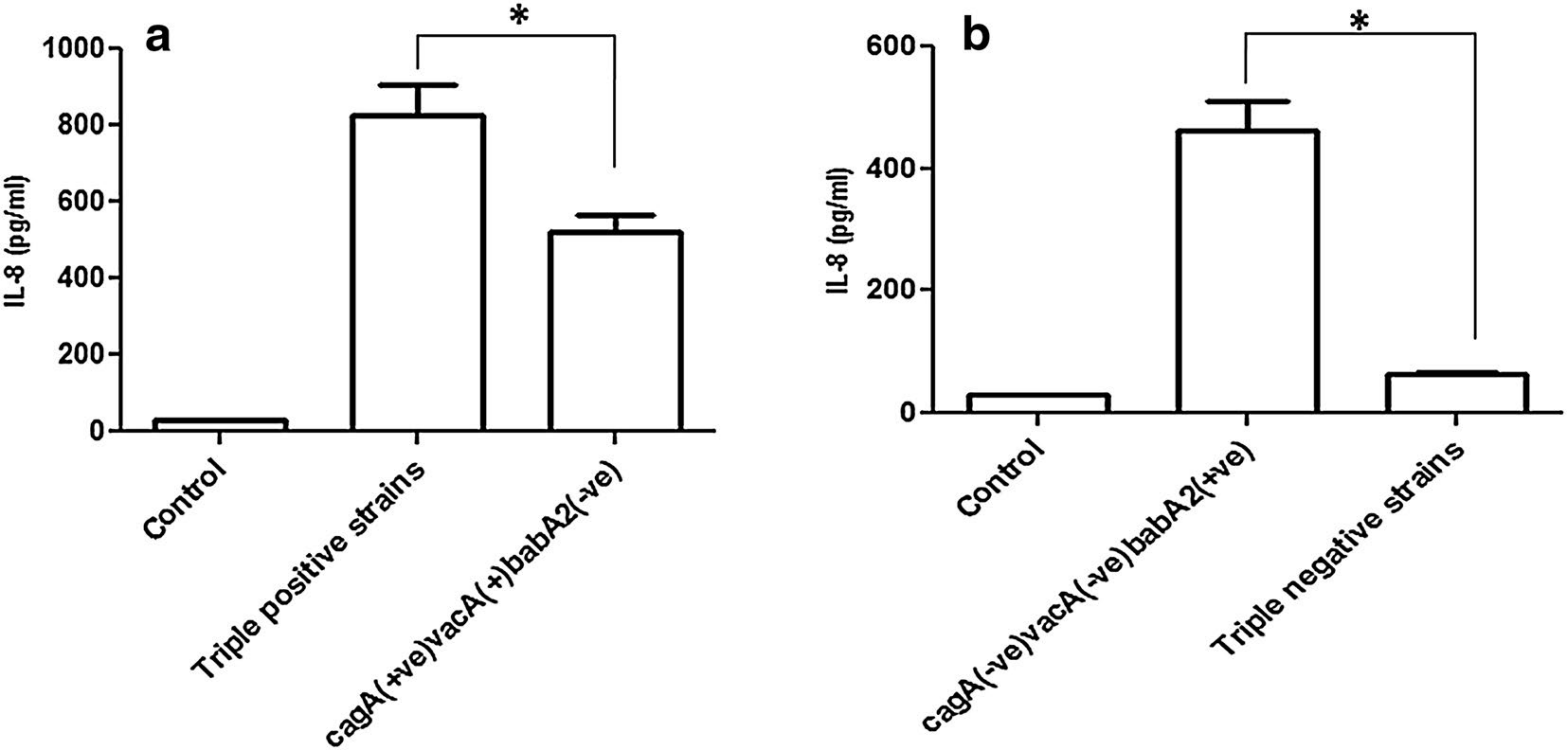
Triple positive strains and even the *babA2* positive only strains enhance IL-8 production in AGS cells. In vitro IL-8 production from AGS cells co-cultured with randomly selected **a** triple positive and *cagA* ^+^/*vacA* ^+^/*babA2* ^−^ strains, and **b** *cagA* ^−^/*vacA* ^−^/*babA2* ^+^ and triple negative *H. pylori* strains (MOI is 100) for 8 h. IL-8 from culture supernatant was measured using ELISA as described in “Methods”. Data are expressed as mean ± standard error of mean (SEM) of 3 experiments in duplicates. *P < 0.05 as compared between groups

Based on the presence or absence of these three virulence genes, the tested strains were divided into four groups: (1) triple positive strains (*cagA*^+^*/vacA*^+^*/babA2*^+^), (2) *cagA*^+^*/vacA*^+^*/babA2*^−^ strains, (3) *cagA*^−^*/vacA*^−^*/babA2*^+^ strains, and (4) triple negative strains (*cag*−*/vac*^−^*/babA2*^−^). After co-cultured with *H. pylori* strains for 8 h the IL-8 secretion was significantly (*P* < 0.05) higher from those cells which were infected with the triple positive strains (827.3 ± 80.88 pg mL^−1^, n = 3) than the cells infected with *cagA*^+^*/vacA*^+^*/babA2*^−^ strains (523.1 ± 43.84 pg mL^−1^, n = 3, Fig. 2a). Similarly, significantly (*P* < 0.05) higher amount of IL-8 induction was recorded when the cells were infected with *cagA*^−^*/vacA*^−^*/babA2*^+^ strains (462.7 ± 47.81 pg mL^−1^, n = 3) compared to triple negative strains (62.80 ± 2.30 pg mL^−1^, n = 3, Fig. 2b).

### *babA2* mutant strains show decreased level of IL-8 induction in AGS cells

**Fig. 3:**
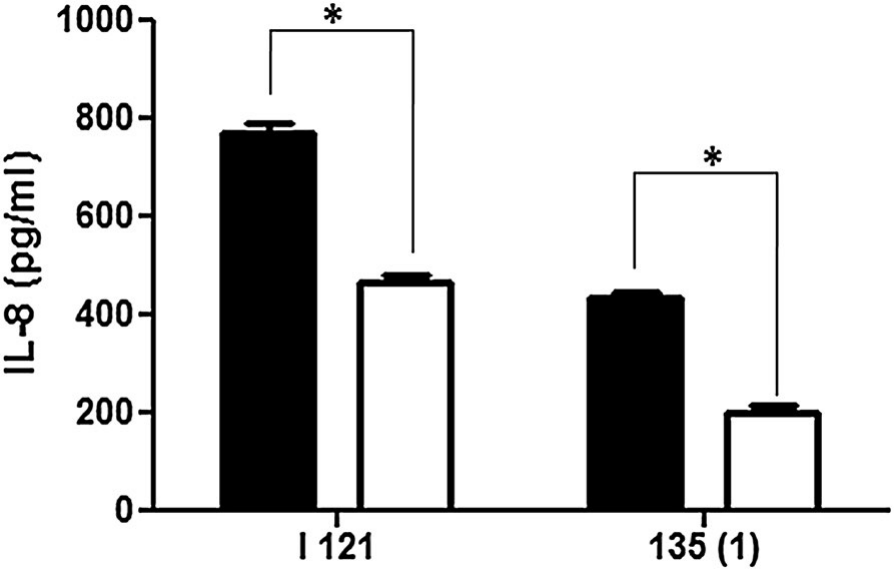
Wild type *H. pylori* strains show higher IL-8 production than their isogenic mutant of *babA2* in AGS cells. IL-8 from culture supernatant was measured using ELISA as described in “Methods”. Data are expressed as mean ± standard error of mean (SEM) of 3 experiments in duplicates. Wild type is denoted with *black filled bar* (◼) and mutants denoted with *empty bar* (◻). *P < 0.05 as compared between groups

To further establish our data we carried out the IL-8 assay in cell culture as described previously by infecting the cells with I-121, 135(1) wild type strains and their *babA2* mutants separately. The results indicated that the significantly (*P* < 0.05) higher amount of IL-8 induction was recorded from the cells infected with the wild type strains (729.18 ± 22.39 and 433.95 ± 12.41 pg mL^−1^ respectively, Fig. 3) rather than its *babA2* mutants (465.06 ± 16.19 and 202.77 ± 12.33 pg mL^−1^ respectively, Fig. 3).

### Triple positive strains trigger more apoptosis in AGS cell line

**Fig. 4:**
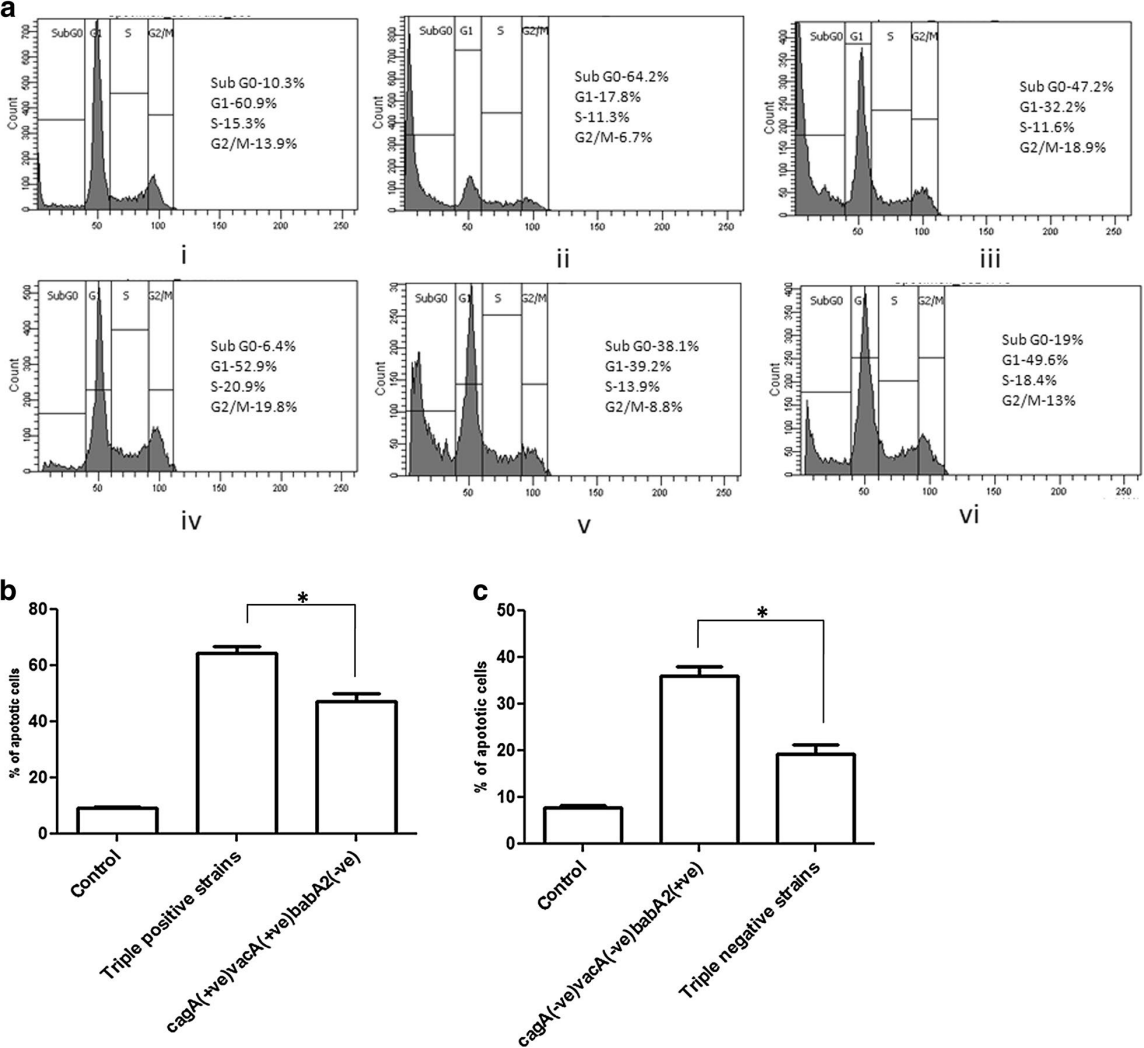
**a**–**c** Triple positive strains and even the *babA2* positive only strains cause more apoptosis in AGS cells. **a** Apoptosis of AGS cells (**a** *i*, *iv* worked as controls) co-cultured with different genotypic variant i.e. **a** *ii* triple positive; **a** *iii* *cagA* ^+^/*vacA* ^+^/*babA2* ^−^; **a** *v* *cagA* ^−^/*vacA* ^−^/*babA2* ^+^; and **a** *vi* triple negative strains of *H. pylori* strains for 24 h (MOI is 100), stained with propidium iodide and analysed by flow cytometry. These *figures* are representative profile of at least three experiments. **b**, **c** Graphical representation of  % apoptotic cells (Sub G0 phase) infected with same group of strains were expressed as mean ± SEM. *P < 0.05 as compared between groups

The cell cycle analysis with propidium iodide reveals distribution of cells in three major phases of the cell cycle (G1 vs. S vs. G2/M, Fig. 4a) and makes it possible to detect unhealthy cells with fractional DNA content. The cells in the sub-G0 phase represent apoptotic cells. After co-culturing the AGS cells with *H. pylori* for 24 h significantly (*P* < 0.05) higher amount of apoptotic cell death was found when the cells infected with triple positive strains (64.40 ± 2.485 %, n = 3, Fig. 4aii, b) in comparison to the cells infected with *cagA*^+^*/vacA*^+^*/babA2*^−^ strains (47.17 ± 2.916 %, n = 3, Fig. 4aiii, b). Similarly, *cagA*^−^*/vacA*^−^*/babA2*^+^ strains (36.17 ± 1.955 %, n = 3, Fig. 4av, c) showed significantly (*P* < 0.05) higher apoptotic cell death than the triple negative strains (19.33 ± 2.038 %, n = 3, Fig. 4avi, c) after 24 h infection.

### Mutation of *babA2* gene causes reduced apoptosis in ex vivo

**Fig. 5:**
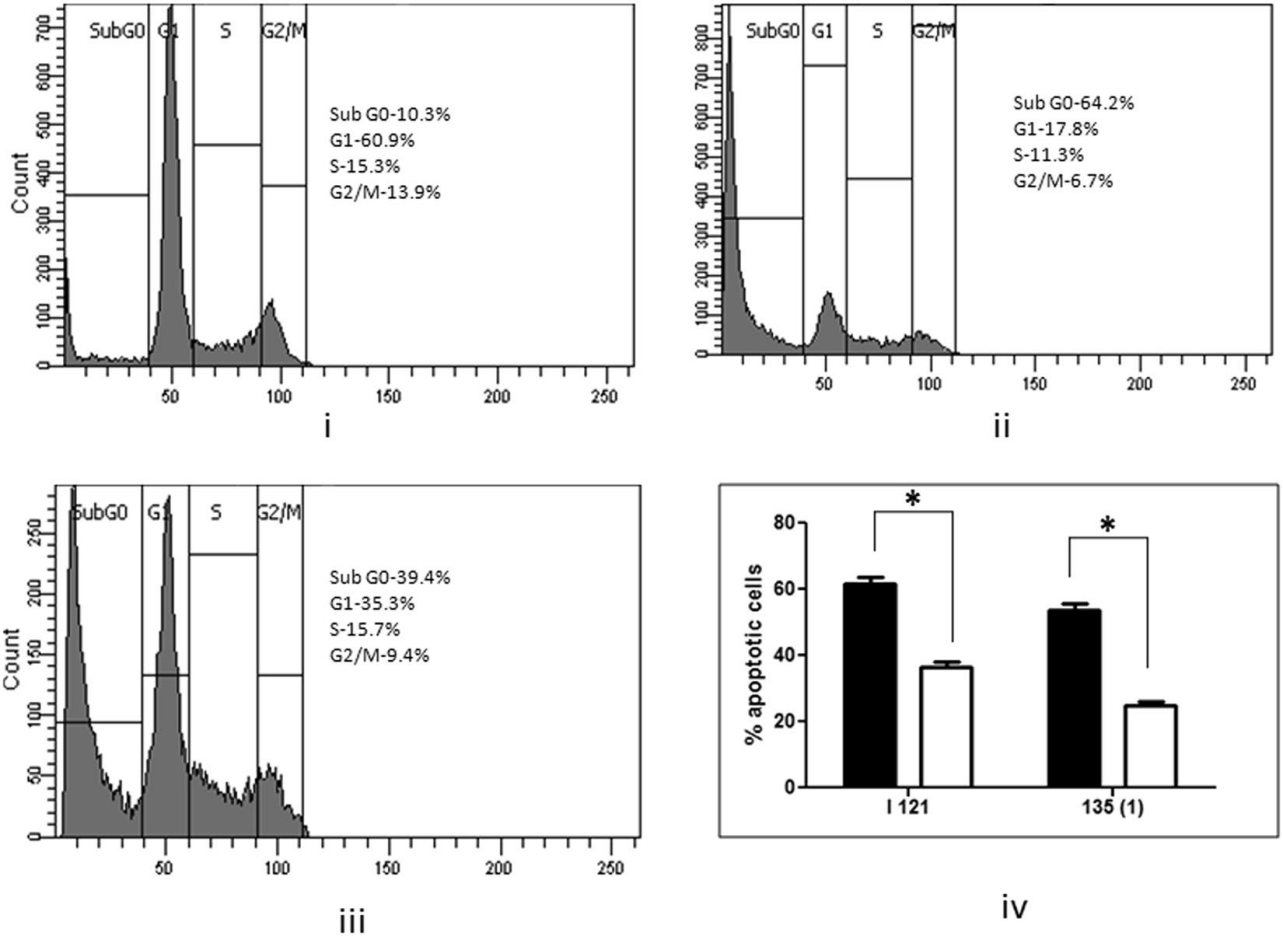
Wild type *H. pylori* strains cause more apoptotic cell death than their isogenic mutant of *babA2* in AGS cells. Apoptosis of AGS cells (**i** worked as controls) co-cultured with different genotypic variant i.e. **ii** wild type I-121 strain; and **iii** isogenic *babA2* mutant of I-121 strain for 24 h (MOI is 100), stained with propidium iodide and analysed by flow cytometry. **i**–**iii** is only representative profile of at least three experiments carried out with I-121 strain and its *babA2* mutant. **iv** Graphical representation of percentage of apoptotic cells (sub G0 phase) infected with two wild type and their *babA2* mutant strains were expressed as mean ± SEM. Wild type is denoted with *black filled bar* (◼) and mutants denoted with *empty bar* (◻). *P < 0.05 as compared between groups

For reconfirming the apoptotic potential of the *babA2* gene we carried out the cell cycle analysis experiment in AGS cells by infecting the cells with two wild type strains [I-121 and 135 (1) respectively] and their *babA2* deletion mutants as described previously. After 24 h of infection it was found that the wild type strains were capable of significantly (*P* < 0.05) more apoptotic cell death (61.8 ± 1.88, 53.56 ± 1.946 % respectively, Fig. 5) than their isogenic mutants of *babA2* (36.43 ± 1.486, 24.76 ± 1.328 %, respectively, Fig. 5).

## Discussion

Various studies have indicated that the incidence and/or severity of gastroduodenal pathologies related to *H. pylori* may vary according to geographical regions [27]. There is also evidence for the existence of different strains of *H*. *pylori* with different degrees of virulence indicating the variation in the distribution of the different virulence attributes of *H. pylori* in different populations [29–31]. The *cagA* gene was found at a frequency of 86 % among the 114 tested strains of this region and this virulence marker was found at almost equal frequencies in strains from DU patients (90.6 %) and NUD patients (82 %), indicating that the prevalence of the *cagA* gene cannot be considered as a key virulence marker for determination of the clinical status of the host, as has been reported in other Indian studies [13, 32]. In our study most of the strains have type A *cagA* without any association in disease outcome. All the strains used in sequence analysis consist of Western-CagA-specific sequence and it is in agreement with our previous report [11]. We also found that highly virulent *s1m1* allele of *vacA* gene was present among 71.9 % of Indian *H. pylori* strains and presence of this allele did not show any correlation with the disease outcome (DU = 73.6 %, NUD = 70.5 %) of the host. This data is in accord with the previous report having 70 % presence of *s1m1* allele in Indian *H. pylori* isolates [5].

Here we found that the Indian *H. pylori* strains exhibited 67.5 % prevalence of *babA2* gene and the *babA2* gene was uniformly distributed (DU = 69.8 %, NUD = 65.6 %) irrespective of disease status. These results indicate that, unlike in the Western countries [33], presence of *babA2* is not associated with clinical outcome in India. In our study among 84 double positive strains, 61 were found as triple positive. These 61 triple positive strains also showed an almost equal distribution in DU (29/53, 54.7 %) patients and NUDs (32/61, 52.4 %), which is inconsistent with the report from few Western countries [34].

More recent analyses of *babA2* as a virulence marker have produced conflicting data on the usefulness of *babA2* expression in predicting clinical outcome, which is most likely dependent on the geographic origin of the *H. pylori* strains. In Portuguese and Thai populations, *babA2* is not a biomarker for peptic ulcer disease or gastric cancer [35, 36]. However, for strains isolated from Germany, Turkey, or northern Portugal, *babA2* expression is associated with the severity of gastric disease [18, 37, 38]. This inconsistency may be due to the specific geographic variation of the circulating bacterial lineages. Another explanation for this lack of association may be due to the allelic variation of the *babA2* gene which was studied by Pride et al. [39].

An inflammatory response is one of the main pathophysiological events in *H. pylori* mediated infection. It has been shown that epithelial cells secrete IL-8 as a result of *H. pylori* infection [40–42]. IL-8 acts as a potent chemo-attractant for neutrophils and is thought to play a crucial role in *H. pylori* induced tissue damage [43]. Several studies have shown that induction of IL-8 secretion from host cells is dependent on the presence of CagA, VacA, OMPs (OipA) and LPS [43–46]. In order to see the effect of *babA2* gene on the IL-8 induction, triple positive (*cagA*^−^/*vacA*^−^/*babA2*^+^) strains and triple negative strains were cultured with AGS cells. The triple positive strains induced the highest amount of IL-8 secretion from AGS cells, and the double negative strains but with *babA2* gene caused significantly higher amount of IL-8 secretion than the triple negative ones (Fig. 2, *P* < 0.05). Therefore, it may be emphasized that the triple positive strains and also the presence of *babA2* somehow enhances the ability of *H. pylori* to induce IL-8 in the gastric mucosa.

IL-8 secretion and inflammation due to *H. pylori* infection lead to epithelial cell damage or apoptosis. Apoptosis is a genetically programmed form of cell death which is mainly characterized by some distinct morphological and molecular features. Microbial pathogens or their products can directly activate the apoptotic pathway which plays a role in pathogenesis [47]. It has been reported that *cagA*, and *vacA* have an effect on *H. pylori* mediated apoptosis [48, 49]. In order to elucidate the role of *babA2* in apoptosis, the triple positive, *cagA*^+^/*vacA*^+^/*babA2*^−^, *cagA*^−^/*vacA*^−^/*babA2*^+^ and triple negative strains were used independently to infect the AGS cells. It was observed that the triple positive strains caused more epithelial cell death than the other strains, whereas the *cagA*^−^/*vacA*^−^/*babA2*^+^ strains caused significantly higher level of apoptosis than the triple negative strains (*P* < 0.05).

As the triple positive strains exhibit highest level of IL-8 induction as well as apoptosis that is why we made isogenic *babA2* mutants of these strains [I-121 and 135(1) respectively] to reconfirm the role of *babA2* in *H. pylori* virulence. The IL-8 induction assay and cell cycle analysis by flow cytometry were performed with these wild type and mutant strains as described in methods section. In agreement, it was found that the wild type strains were significantly (*P* < *0.05*) more capable of IL-8 induction as well as mediating the apoptotic cell death in AGS cells (Figs. 3, 5). These data more strongly establish the virulence potential of the strains with *babA2* positive genotype.

It has been already reported that ex vivo IL-8 production requires the adherence of viable *H. pylori* to the AGS cells [50], and as the induction of apoptosis and IL-8 secretion are often linked [51], so it can be said that the apoptosis in AGS cells also require the adherence of *H. pylori*. In this study, it was found that the adherence of *babA2* positive and *babA2* negative strains were comparable in AGS cells. In agreement, it was also found that the extent of adherence to the AGS cells were comparable between the wild type strains and isogenic *babA2* mutants. This report suggests that although adherence is crucial, the *babA2* gene products may regulate the IL-8 secretion and apoptosis by an adherence independent manner. *H. pylori* has at least five different adhesin factors which use different receptors for adhering on gastric epithelial cells [43], suggesting that presence or absence of only one of these genes may not affect its adherence to gastric cells. Further studies will be required to explain what other factors may impact the extent of adherence.

In this report, although it was observed that Indian *H. pylori* strains harbored *babA2* gene independent of disease status and also the triple positive strains showed no association with disease status, but on the other hand, the triple positive strains and even only the *babA2* positive strains showed more virulence as they induced more IL-8 secretion and as well as caused more apoptosis in AGS cell line than the triple negative strains. In accordance, the wild type strains also exhibited more capability to induce IL-8 secretion as well as apoptosis than their isogenic *babA2* mutants in the cell culture study. Apparently, it seems that these two statements are contradictory. What could be the probable reasons? *H. pylori* is one of the genetically diverse bacterial species with regard to genotyping [52–54]. Previous reports also suggested that *H. pylori* genotype varies geographically [2, 54]. The polymorphisms of some cytokine genes have been found to be associated with *H. pylori* mediated clinical consequences, probably because they modulate the amount of cytokine production in response to *H. pylori* infection [55]. It was already been established that dietary salt intake can play an important role in enhancing the likelihood of severe clinical consequences of *H. pylori* mediated infection [56]. Environmental iron level also brings about the changes in the composition of the *H. pylori* outer membrane which ultimately influences the pattern of clinical outcomes [57]. These above mentioned information clearly establish the role of host factors and environmental factors in the disease outcomes of *H. pylori* mediated infection. Importantly, *H. pylori* toxin gene expression is inconsistent with the general phenomenon observed for other pathogenic bacteria, that is, that expression of the important toxin genes are strongly associated with diseases [58]. The expressions of toxin genes by *H.pylori* are not solely dependent on the disease status. So, this result can not lead to the underestimation of the role of *babA2* gene as a toxin gene. The genetic constitution of host may play a critical role in the successful colonization and ultimately in the disease consequences of *H. pylori* infection. We can say that the virulent effect of *babA2* gene of *H. pylori* may come into action only after interplay with certain host factors. Our results also showed that *babA2* gene in combination with the presence of *cagA* and *vacA s1m1* allele become much more toxic as the triple positive strains showed highest degree of IL-8 induction as well as apoptotic cell death in cell culture study, although the PCR-based genotyping results showed a uniform distribution of triple positive strains independent of disease status. These discrepancies may be due to the fact that at the time of *H. pylori* isolation, the NUD people harboring the triple positive strains may be at the risk of developing an ulcer disease in future which cannot be come into our count.

## Conclusion

In conclusion, although the presence of *babA2* gene is not associated with disease outcome in India, but the *babA2* positive strains showed more virulence than their negative counterparts in ex vivo study. Study also indicated that adherence of *H. pylori* to the gastric epithelial (AGS) cells is not solely dependent on *babA2.* It is evident from this study that bacterial genetic constitution cannot be considered as only responsible factor for disease consequences. Certain host factors along with bacterial genetic traits may play a crucial role in this aspect. Further studies are required to determine the function(s) of *babA2* and its relationship with disease outcomes.

## Methods

### Collection of biopsy samples

Biopsy specimens were obtained as described previously [58] from a total of 185 adult subjects with upper gastrointestinal irregularities underwent endoscopy at the hospital of the Institute of Post Graduate Medical Education and Research, Kolkata, and St John’s Medical College Hospital, Bangalore, India, during 2009–2011. A detailed case study of each individual was done prior to endoscopy. The objective of the study was explained to each individual and informed consent was obtained from each of them under protocols approved by the ethical committees of respective institutes based on the Helsinki Declaration. These biopsy samples were transported to the laboratory under ice-cold condition in *Brucella* broth (0.6 ml, Difco Laboratories) with glycerol (15 %) and stored in −70 °C until culture.

### *Helicobacter pylori* culture

The *Brucella* broth containing the biopsy samples were vortexed in laboratory for 2 min and 200 µl of the mixture was streaked onto brain heart infusion agar (BHIA) plate containing horse serum (7 %, Invitrogen, Grand Island, NY, USA), IsoVitalex (0.4 %, Becton–Dickinson, San Jose, CA, USA), trimethoprim (5 µg mL^−1^), vancomycin (6 µg mL^−1^), polymyxin B (10 µg mL^−1^) and nalidixic acid (8 µg mL^−1^) (all from Sigma Chemicals, MO, USA). These plates were incubated at 37 ^°^C in microaerophilic condition (85 % N_2_, 10 % CO_2_, and 5 % O_2_) for 3–6 days [10] in a double gas incubator (Heraeus Instruments, Hanau, Germany) and were identified on the basis of their typical morphology and urease, oxidase and catalase test result.

### DNA extraction and genotyping of *cagA, vacA* and *babA2*

Genomic DNA was extracted by using CTAB method with phenol/chloroform and isopropanol precipitation as described elsewhere [59]. Purified DNAs were stored at −20 ℃ until use. All the PCR reactions were carried out in a 25 µl reaction volume containing genomic DNA (50 ng), forward and reverse primers (25 pmol), each deoxynucleoside triphosphate (0.25 mM each, Roche, Berlin, Germany), Taq DNA polymerase (1 U, Genei, Bangalore, India), and Mgcl_2_ (1.5 mM) in a standard PCR buffer (Genei, Bangalore, India) for 35 cycles, generally under the following reaction condition: 95 ℃ for 1 min, 55 ℃ for 1 min, and 72 ℃ for a time chosen based on the size of the expected amplified fragment (1 min kb^−1^) in a Master Cycler apparatus (Eppendorf, Hamburg, Germany). The primers used in this study are listed in Table 4.

**Table 4:** Primers used in this study for PCR amplification

Primer	Sequence (5′–3′)	Amplicon (bp)	Reference
nthu_babA2F babA2R babA2F babA2R607 UreBF UreBR CagA5cF CagA3cR vacAsF vacAsR vacAmF vacAmR	AATCGAAAAAGGAGAAAACATGAAAAA TGTTAGTGATTTCGGTGTAGGACA AATCCAAAAAGGAGAAAAAGTATGAAA GTTTTCTTTGAGCGCGGGTAAGC CGTCCGGCAATAGCTGCCATAGT GTAGGTCCTGCTACTGAAGCCTTA GTTGATAACGCTGTCGCTTCA GGGTTGTATGATATTTTCCATAA ATGGAAATACAACAAACACAC CTGCTTGAATGCGCCAAAC CAATCTGTCCAATCAAGCGAG GCGTCAAAATAATTCCAAGG	832 607 480 350 *s1*-259/*s2*-286 *m1*-567/*m2*-642	[26] [20] [20] [61] [5] [5] [62] [62] [63] [63] [63] [63]

### Generation of *babA2* mutant *H. pylori*

A 832 bp fragment of *babA2* was cloned into TOPO TA cloning vector (Invitrogen, USA) to generate the plasmid pcr2.1: *babA2*. The isolated plasmid (pCR2.1: *babA2*) DNA was digested with restriction enzyme *BsmI* (New England Biolabs, USA) and ligated with chloramphenicol cassette amplified from DR2 plasmid by phusion enzyme (Thermo-scientific) to produce plasmid pcr2.1: *babA2*: cmp. This construct was then used to electroporate *babA2* positive *H. pylori* cells. Transformed single colonies were sub cultured and screened for the interruption of *babA2* gene by PCR with babA2F/babA2R set of primers. Transformed colonies (positive colonies) gave about 1.6 kb amplicon rather than 832 bp.

### IL-8 assay

All the bacterial strains were cultured in 7 % serum containing BHIA plates for 24 h at 37 °C under microaerophilic conditions. In order to obtain ex vivo IL-8 secretion from gastric epithelial cells, AGS **(**human gastric adenocarcinoma cell line) cells were plated (2.5 × 10^5^ cells mL^−1^) into 24 well plates and cultured for 24 h. *H. pylori* [multiplicity of infection (MOI) of 100] were added to cultured cells. After 8 h of infection, IL-8 levels in the supernatant were assayed in duplicate three times using a commercially available specific ELISA kit (Genetix, New Delhi, India) following the manufacturer’s protocols.

### Cell cycle analysis

AGS cells (1 × 10^6^ cells mL^−1^ in each well) were infected with 1 day old *H. pylori* culture. After 24 h of infection cells were fixed in 70 % chilled ethanol and were kept at 4 ℃ for further analysis. Prior to analysis cells were washed in 2 % fetal bovine serum (FBS) containing PBS (pH 7.4) and the cell pellets were stained with propidium iodide (50 µg mL^−1^) containing DNase-free RNase (0.1 mg mL^−1^). Cells were then acquired on flow cytometer and the data was analyzed in FACS Diva software (Becton–Dickinson, USA).

### Adherence assay

AGS cells were cultured to about 70–80 % confluency in complete RPMI-1640 medium. AGS cells were washed three times with sterile PBS, and RPMI with 10 % FBS was added. 24 h old *H. pylori* culture was washed with sterile PBS and suspended in incomplete RPMI. Adherence assay was performed by adding the bacterial cells to the AGS monolayer (MOI 100) and incubated for 2 h [60]. After 2 h of infection unadhered bacterial cells were removed and washed three times with sterile PBS. The adhered bacteria were collected in PBS by scrapping the cell-line along with the infected bacteria. The CFU was determined by plating various serial dilutions of these bacterial suspensions on BHI agar plates. All experiments were repeated at least thrice for each strain.

### Statistical analysis

Each experiment was performed at least thrice in duplicates and results expressed as mean ± standard error of the mean (SEM). Statistical analysis was done by T test and ANOVA (wherever applicable), using Graph Pad Prism software (version 5, Graph Pad Software Inc, La Jolla, CA, USA), *P* values <0.05 were considered to be significant.
